# Serum Lipopolysaccharide-binding Protein Levels and the Incidence of Metabolic Syndrome in a General Japanese Population: the Hisayama Study

**DOI:** 10.2188/jea.JE20220232

**Published:** 2024-01-05

**Authors:** Shoko Tomooka, Emi Oishi, Masako Asada, Satoko Sakata, Jun Hata, Sanmei Chen, Takanori Honda, Kosuke Suzuki, Hiroshi Watanabe, Norihito Murayama, Naohisa Wada, Takanari Kitazono, Toshiharu Ninomiya

**Affiliations:** 1 Department of Epidemiology and Public Health, Graduate School of Medical Sciences, Kyushu University, Fukuoka, Japan; 2 Department of General Dentistry, Graduate School of Dental Sciences, Kyushu University, Fukuoka, Japan; 3 Department of Medicine and Clinical Science, Graduate School of Medical Sciences, Kyushu University, Fukuoka, Japan; 4 Department of Anesthesiology and Critical Care Medicine, Graduate School of Medical Sciences, Kyushu University, Fukuoka, Japan; 5 Center for Cohort Studies, Graduate School of Medical Sciences, Kyushu University, Fukuoka, Japan; 6 Global Health Nursing, Graduate School of Biomedical and Health Sciences, Hiroshima University, Hiroshima, Japan; 7 Research Institute, Suntory Global Innovation Center Ltd, Kyoto, Japan; 8 Department of General Dentistry, Faculty of Dental Science, Kyushu University, Fukuoka, Japan

**Keywords:** metabolic syndrome, endotoxemia, epidemiology, follow-up studies, lipopolysaccharide-binding protein

## Abstract

**Background:**

The association between chronic lipopolysaccharide exposure and the development of metabolic syndrome (MetS) is unclear. In this study we examined the association between serum lipopolysaccharide-binding protein (LBP) levels, an indicator of lipopolysaccharide exposure, and the development of MetS in a general Japanese population.

**Methods:**

1,869 community-dwelling Japanese individuals aged ≥40 years without MetS at baseline examination in 2002–2003 were followed up by repeated examination in 2007–2008. MetS was defined according to the Japanese criteria. Serum LBP levels were classified into quartiles (quartiles 1–4: 2.20–9.56, 9.57–10.78, 10.79–12.18, and 12.19–24.34 µg/mL, respectively). Odds ratios (ORs) for developing MetS were calculated using a logistic regression model.

**Results:**

At the follow-up survey, 159 participants had developed MetS. Higher serum LBP levels were associated with greater risk of developing MetS after multivariable adjustment for age, sex, smoking, drinking, and exercise habits (OR [95% confidence interval] for quartiles 1–4: 1.00 [reference], 2.92 [1.59–5.37], 3.48 [1.91–6.35], and 3.86 [2.12–7.03], respectively; *P* for trend <0.001). After additional adjustment for homeostasis model assessment of insulin resistance, this association was attenuated but remained significant (*P* for trend = 0.007). On the other hand, no significant association was observed after additional adjustment for serum high-sensitivity C-reactive protein (*P* for trend = 0.07).

**Conclusion:**

In the general Japanese population, our findings suggest that higher serum LBP levels are associated with elevated risk of developing MetS. Low-grade endotoxemia could play a role in the development of MetS through systemic chronic inflammation and insulin resistance.

## INTRODUCTION

Metabolic syndrome (MetS) is defined as a pathologic condition characterized by abdominal obesity, dyslipidemia, impaired glucose tolerance, and elevated blood pressure. Individuals with MetS have an elevated risk of developing type 2 diabetes and cardiovascular disease.^1^^–^^3^ Chronic low-grade inflammation is an established factor in the etiopathogenesis of obesity and insulin resistance, which are intimately related to the metabolic disorders observed in MetS.^4^ However, the contributing factors by which chronic low-grade inflammation could increase the risk of MetS have not yet been clarified. Lipopolysaccharide, a major component of the outer membrane of Gram-negative bacteria, has been shown to stimulate the release of several pro-inflammatory cytokines, which could eventually induce insulin resistance.^5^ The condition of chronically elevated serum levels of lipopolysaccharide is also known as “metabolic endotoxemia”.^6^ Recent studies in mice^6^^–^^8^ and humans^9^ suggest that this condition is linked with obesity, insulin resistance, and diabetes mellitus, even though the serum concentrations of lipopolysaccharide in metabolic endotoxemia are 10–50 times lower than those in sepsis.^6^ Thus, it has been hypothesized that lipopolysaccharide is a triggering factor of metabolic abnormalities.

Lipopolysaccharide-binding protein (LBP) is an acute-phase protein that is synthesized mainly in the liver.^10^ LBP can be detected in the blood of even healthy individuals following exposure to indigenous bacteria.^11^^,^^12^ LBP has been reported to promote the activation of an inflammatory response when lipopolysaccharide enters the blood even at a subclinical level of inflammation.^13^^,^^14^ Binding of the LBP–lipopolysaccharide complex to the cluster of differentiation 14, which is mainly expressed by macrophages and neutrophils, has been shown to mediate transduction signals, including nuclear factor kappa B activation via toll-like receptor 4 (TLR4), leading to the activation of inflammatory responses.^15^^,^^16^ It has been reported that serum LBP concentrations reach a peak within 12 hours after a small amount of lipopolysaccharide exposure,^17^ and LBP has a longer half-life than lipopolysaccharide in blood.^14^^,^^17^ Therefore, the serum LBP concentration is a good marker of endotoxemia.^14^^,^^18^^,^^19^ Several epidemiological studies have reported cross-sectional associations of serum LBP levels with the presence of MetS.^11^^,^^12^^,^^20^^,^^21^ However, there has been only one population-based longitudinal study addressing the association of serum LBP levels with the development of MetS.^22^

The Hisayama Study is an ongoing population-based prospective cohort study of cardiovascular disease and lifestyle-related diseases that has been conducted since 1961 in the town of Hisayama, a suburb of the Fukuoka metropolitan area in Japan.^23^ This study previously reported that the serum LBP concentration was strongly correlated with the serum high-sensitivity C-reactive protein (hs-CRP), a marker of low-grade systemic chronic inflammation, and that higher serum LBP levels were associated with increased risk of developing cardiovascular disease through chronic systemic inflammation in a community-dwelling Japanese population.^24^ In the present study, we investigated the association of serum LBP levels with the development of MetS using prospective longitudinal data from the same community-dwelling Japanese population, accounting for the potential mediating effect of chronic systemic inflammation and insulin resistance in the association.

## METHODS

### Study participants and follow-up survey

**Figure 1.**
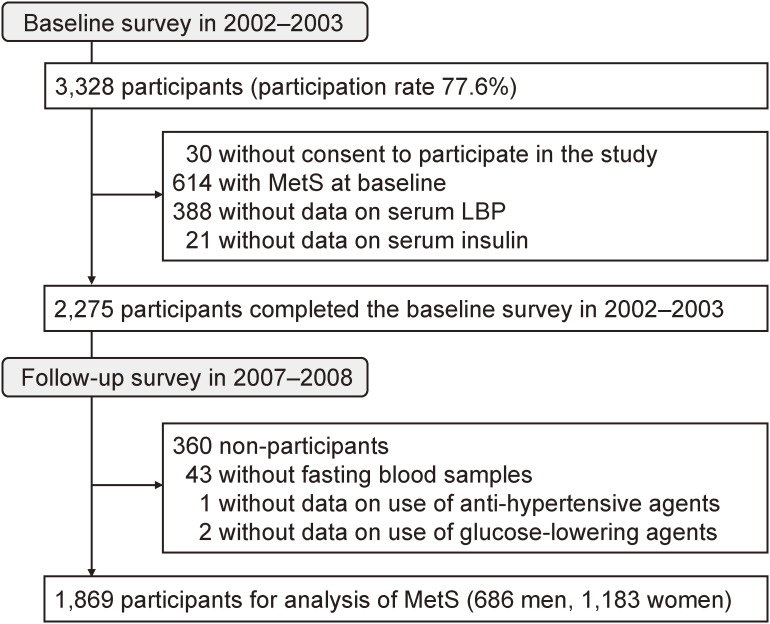
Selection process of the examined population. LBP, lipopolysaccharide-binding protein; MetS, metabolic syndrome.

In 2002–2003, the baseline screening survey for the present study was administered to a total of 3,328 residents aged 40 years or older (participation rate: 77.6%) of the town of Hisayama. After excluding 30 individuals who did not consent to participate in the study, 614 individuals with MetS at baseline, 388 individuals without available data on serum LBP concentrations, and 21 individuals without data on serum insulin, 2,275 individuals who completed the baseline survey remained. After excluding 360 individuals who did not participate the follow-up survey in 2007–2008 (including 159 individuals who died during the follow-up period), 46 individuals who were missing data on one or more components of MetS in 2007–2008, the remaining 1,869 individuals (686 men and 1,183 women) were included in the present study (Figure 1). This study was approved by the Kyushu University Institutional Review Board for Clinical Research, and written informed consent was obtained from all participants.

### Measurement of serum lipopolysaccharide-binding protein concentrations

Serum specimens collected at the baseline screening survey were stored at −80°C until used for the measurements of serum LBP concentrations in 2018. Serum LBP concentrations were measured using enzyme-linked immunosorbent assay kits (Hycult Biotech; catalog number HK315) in accordance with the manufacturer’s instructions. The participants were divided into four categories according to the quartiles of serum LBP levels (quartile 1: 2.20–9.56 µg/mL; quartile 2: 9.57–10.78 µg/mL; quartile 3: 10.79–12.18 µg/mL; quartile 4: 12.19–24.34 µg/mL).

### Definition of metabolic syndrome

The criteria for MetS in this study were defined based on those proposed by the Japanese Society of Internal Medicine (Japanese criteria).^25^ The detailed criteria are shown in eMaterial 1. In addition, in the sensitivity analyses using various MetS criteria, we used the modified Japanese criteria^26^ where the cutoff values of waist circumference for Asians^27^ (≥90 cm in men and ≥80 cm in women), the criteria of the International Diabetes Federation (IDF) for Asians,^27^ the European Group for the Study of Insulin Resistance criteria,^28^ and the criteria in the joint statement from the IDF, the National Heart, Lung, and Blood Institute, the American Heart Association, the World Heart Federation, the International Atherosclerosis Society, and the International Association for the Study of Obesity^1^ (eTable 1).

### Clinical evaluation and laboratory measurements

Detailed information on the clinical evaluations and laboratory measurements is provided in eMaterial 1.

### Statistical analyses

Serum triglycerides, homeostasis model assessment of insulin resistance (HOMA-IR), and serum hs-CRP were logarithmically transformed because of skewed distributions. The trends in the means and the frequencies of risk factors across the quartiles of serum LBP levels were tested using a linear and a logistic regression analysis, respectively. The age- and sex-adjusted cumulative incidence of MetS was calculated using the direct method. We used the logistic regression model to estimate the odds ratios (ORs) and 95% confidence intervals (CIs) of the development of MetS according to the quartiles of serum LBP levels as well as per 1-standard deviation (SD) increments of the serum LBP concentrations. In this analysis, the risk estimates were calculated in four different models including the covariates at baseline: (1) an age- and sex-adjusted model; (2) model 1, adjusted for age, sex, current smoking, current drinking, and regular exercise; (3) model 2, adjusted for the covariates included in model 1 plus HOMA-IR; and (4) model 3, adjusted for the covariates included in model 1 plus serum hs-CRP. We also examined the mediation effects of HOMA-IR or serum hs-CRP on the association between serum LBP concentration and MetS development using the SAS procedure PROC CAUSALMED. The heterogeneity in the association between subgroups was tested by adding multiplicative interaction terms to the relevant logistic model. For the sensitivity analyses, we performed the analysis after excluding individuals with serum hs-CRP of ≥3.0 mg/L to account for the influence of acute inflammations.

Moreover, we investigated the associations of serum LBP levels with the development of each MetS component using logistic regression models including the same sets of covariates. The analyses for each outcome (ie, abdominal obesity, elevated blood pressure, elevated fasting plasma glucose, and dyslipidemia) were conducted by excluding those who had each component at baseline. The selection process of the examined population for each component of MetS is shown in eFigure 1. We also estimated the association between serum LBP levels at the baseline survey and HOMA-IR at the follow-up survey.^29^ All statistical analyses were performed using the SAS statistical software program, version 9.4 (SAS Institute Inc., Cary, NC, USA). A two-sided value of *P* < 0.05 was considered statistically significant in all analyses.

## RESULTS

The baseline characteristics of the study population according to the quartiles of serum LBP levels are summarized in Table 1. The mean values of age, systolic blood pressure, diastolic blood pressure, fasting plasma glucose, serum total cholesterol, body mass index, and waist circumference, the median values of serum triglyceride, HOMA-IR, and serum hs-CRP, and the proportions of men, hypertension, use of anti-hypertensive agents, diabetes mellitus, and use of lipid-modifying agents increased significantly with higher serum LBP levels. Meanwhile, higher serum LBP levels were significantly associated with lower mean values of serum high-density lipoprotein (HDL) cholesterol.

**Table 1.** Baseline characteristics of the study population according to serum LBP quartiles, the Hisayama Study, 2002–2003

Variables	Serum LBP levels, µg/mL				*P* for trend

	Q1	Q2	Q3	Q4	

	2.20–9.56	9.57–10.78	10.79–12.18	12.19–24.34	
	(*n* = 462)	(*n* = 471)	(*n* = 469)	(*n* = 467)	
Age, years, mean (SD)	56.8 (11.1)	59.5 (11.2)	62.0 (11.4)	63.7 (11.7)	<0.001
Men, %	34.0	32.5	38.6	41.8	0.003
Hypertension, %	23.8	33.1	42.6	44.5	<0.001
Use of anti-hypertensive agents, %	12.1	17.4	25.4	26.1	<0.001
Systolic blood pressure, mm Hg, mean (SD)	123 (19)	128 (20)	131 (20)	132 (19)	<0.001
Diastolic blood pressure, mm Hg, mean (SD)	74 (10)	77 (11)	78 (11)	78 (11)	<0.001
Diabetes mellitus, %	6.5	8.5	14.3	13.5	<0.001
Use of glucose-lowering agents, %	2.8	2.3	4.5	4.1	0.12
Fasting plasma glucose, mmol/L, mean (SD)	2.6 (0.4)	2.7 (0.4)	2.8 (0.5)	2.8 (0.6)	<0.001
Use of lipid-modifying agents, %	6.3	7.9	11.1	9.2	0.04
Serum total cholesterol, mmol/L, mean (SD)	5.1 (0.8)	5.4 (0.9)	5.3 (1.0)	5.3 (0.9)	0.002
Serum HDL cholesterol, mmol/L, mean (SD)	1.8 (0.4)	1.7 (0.4)	1.6 (0.4)	1.6 (0.4)	<0.001
Serum triglyceride, mmol/L, median (interquartile range)	2.0 (1.5–2.8)	2.4 (1.8–3.3)	2.6 (1.8–3.6)	2.6 (1.8–3.5)	<0.001
Body mass index, kg/m^2^, mean (SD)	21.7 (2.5)	22.5 (2.8)	23.0 (3.1)	23.2 (3.1)	<0.001
Waist circumference, cm, mean (SD)	77.3 (7.8)	80.1 (8.2)	81.2 (8.2)	82.6 (8.6)	<0.001
Current smoking, %	22.5	17.6	19.8	22.5	0.79
Current drinking, %	44.4	43.4	45.2	39.8	0.25
Regular exercise, %	10.0	11.5	10.0	11.1	0.75
HOMA-IR, median (interquartile range)	1.4 (1.0–1.9)	1.5 (1.1–2.2)	1.7 (1.2–2.4)	1.7 (1.2–2.6)	<0.001
Serum hs-CRP, mg/L, median (interquartile range)	0.2 (0.1–0.3)	0.3 (0.2–0.6)	0.5 (0.3–0.9)	1.0 (0.5–2.5)	<0.001

HDL, high-density lipoprotein; HOMA-IR, homeostasis model assessment of insulin resistance; hs-CRP, high-sensitivity C-reactive protein; IQR, interquartile range; LBP, lipopolysaccharide-binding protein; SD, standard deviation.

**Figure 2.**
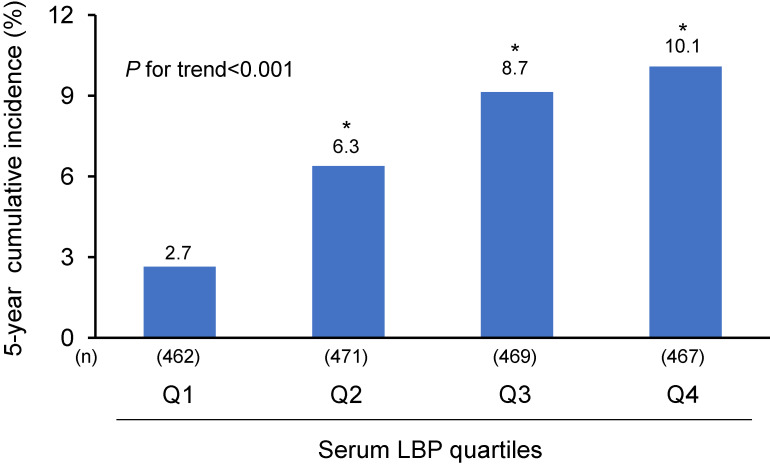
The age- and sex-adjusted 5-year cumulative incidence of MetS according to quartiles of serum LBP levels. Q1 to Q4 indicate ascending quartiles of serum LBP levels: Q1, 2.20–9.56 µg/mL; Q2, 9.57–10.78 µg/mL; Q3, 10.79–12.18 µg/mL; and Q4, 12.19–24.34 µg/mL. ^*^P < 0.001 vs Q1 of serum LBP levels. LBP, lipopolysaccharide-binding protein; MetS, metabolic syndrome; Q, quartile.

At the end of the 5-year follow-up, a total of 159 participants (84 men and 75 women) developed MetS. The age- and sex-adjusted 5-year cumulative incidence of MetS increased significantly with higher serum LBP levels (*P* for trend <0.001) (Figure 2). As shown in Table 2, the ORs of MetS increased significantly with higher serum LBP levels (*P* for trend <0.001) in the multivariable-adjusted model for potential confounders (model 1): the OR was 1.37 (95% CI, 1.17–1.60) per 1-SD increment in the serum LBP concentrations. To address whether insulin resistance or systemic chronic inflammation was involved in the association between serum LBP levels and the risk of MetS, we added either HOMA-IR or serum hs-CRP as an additional covariate to model 1. As a result, the association was attenuated after additional adjustment for HOMA-IR (model 2, *P* for trend = 0.007), but remained significant with an OR of 1.25 (95% CI, 1.06–1.48) per 1-SD increment in the serum LBP concentrations. On the other hand, the additional adjustment for serum hs-CRP in model 1 attenuated the association to a nonsignificant level (*P* for trend = 0.07) with an OR of 1.06 (95% CI, 0.87–1.30) per 1-SD increment in the serum LBP concentrations. We also calculated the direct and indirect effect and proportion mediated by applying the mediation analysis. The proportion of the association between serum LBP concentration and MetS, 35.0% was mediated by HOMA-IR (direct association: OR 1.25; 95% CI, 1.04–1.46; indirect association: OR 1.11; 95% CI, 1.06–1.16). On the other hand, 81.7% was mediated by serum hs-CRP (direct association: OR 1.06; 95% CI, 0.85–1.27; indirect association: OR 1.27; 95% CI, 1.12–1.42) (eTable 2).

**Table 2.** Odds ratios for the development of metabolic syndrome and its components according to serum LBP quartiles

Serum LBP levels	Persons at risk	Number of events	Odds ratio (95% confidence interval)			

			Age- and sex-adjusted	Model 1^b^	Model 2^c^	Model 3^d^
**Metabolic syndrome**						
Q1 (2.20–9.56 µg/mL)	462	15	1.00 (reference)	1.00 (reference)	1.00 (reference)	1.00 (reference)
Q2 (9.57–10.78 µg/mL)	471	41	2.94 (1.60–5.40)	2.92 (1.59–5.37)	2.42 (1.30–4.48)	2.55 (1.38–4.71)
Q3 (10.79–12.18 µg/mL)	469	49	3.49 (1.91–6.36)	3.48 (1.91–6.35)	2.62 (1.42–4.83)	2.61 (1.41–4.84)
Q4 (12.19–24.34 µg/mL)	467	54	3.86 (2.12–7.02)	3.86 (2.12–7.03)	2.61 (1.41–4.82)	2.18 (1.12–4.24)
*P* for trend			<0.001	<0.001	0.007	0.07
Per 1-SD increment in serum LBP concentration^a^			1.37 (1.17–1.59)	1.37 (1.17–1.60)	1.25 (1.06–1.48)	1.06 (0.87–1.30)
**Metabolic syndrome components**						
**Abdominal obesity**						
Q1 (2.20–9.41 µg/mL)	389	50	1.00 (reference)	1.00 (reference)	1.00 (reference)	1.00 (reference)
Q2 (9.42–10.65 µg/mL)	389	69	1.50 (1.01–2.24)	1.52 (1.02–2.27)	1.37 (0.91–2.06)	1.37 (0.92–2.05)
Q3 (10.66–11.99 µg/mL)	391	90	2.13 (1.44–3.14)	2.14 (1.45–3.15)	1.82 (1.22–2.70)	1.68 (1.12–2.51)
Q4 (12.00–24.34 µg/mL)	391	95	2.24 (1.52–3.31)	2.25 (1.52–3.31)	1.77 (1.19–2.64)	1.39 (0.89–2.17)
*P* for trend			<0.001	<0.001	0.003	0.11
Per 1-SD increment in serum LBP concentration^a^			1.31 (1.15–1.48)	1.31 (1.15–1.48)	1.23 (1.08–1.40)	1.06 (0.90–1.24)
**Dyslipidemia**						
Q1 (2.20–9.47 µg/mL)	387	50	1.00 (reference)	1.00 (reference)	1.00 (reference)	1.00 (reference)
Q2 (9.48–10.70 µg/mL)	388	82	1.71 (1.16–2.51)	1.71 (1.16–2.51)	1.59 (1.07–2.34)	1.60 (1.08–2.37)
Q3 (10.71–12.16 µg/mL)	387	88	1.76 (1.19–2.59)	1.76 (1.19–2.59)	1.52 (1.02–2.25)	1.52 (1.01–2.27)
Q4 (12.17–24.34 µg/mL)	388	94	1.84 (1.25–2.71)	1.84 (1.25–2.71)	1.50 (1.00–2.23)	1.38 (0.88–2.17)
*P* for trend			0.005	0.005	0.11	0.26
Per 1-SD increment in serum LBP concentration^a^			1.14 (1.01–1.29)	1.14 (1.01–1.30)	1.07 (0.94–1.21)	0.99 (0.85–1.17)
**Elevated blood pressure**						
Q1 (2.20–9.41 µg/mL)	277	66	1.00 (reference)	1.00 (reference)	1.00 (reference)	1.00 (reference)
Q2 (9.42–10.65 µg/mL)	281	81	1.17 (0.80–1.72)	1.14 (0.78–1.68)	1.07 (0.72–1.58)	1.10 (0.75–1.63)
Q3 (10.66–11.99 µg/mL)	280	82	1.15 (0.78–1.69)	1.13 (0.77–1.66)	1.00 (0.68–1.49)	1.04 (0.70–1.56)
Q4 (12.00–24.34 µg/mL)	281	109	1.65 (1.13–2.41)	1.66 (1.13–2.42)	1.46 (0.99–2.16)	1.46 (0.91–2.18)
*P* for trend			0.01	0.01	0.07	0.19
Per 1-SD increment in serum LBP concentration^a^			1.15 (1.01–1.31)	1.16 (1.02–1.33)	1.12 (0.98–1.28)	1.07 (0.91–1.26)
**Elevated fasting plasma glucose**						
Q1 (2.20–9.48 µg/mL)	378	13	1.00 (reference)	1.00 (reference)	1.00 (reference)	1.00 (reference)
Q2 (9.49–10.72 µg/mL)	375	38	3.22 (1.68–6.18)	3.22 (1.68–6.17)	3.05 (1.59–5.87)	3.13 (1.62–6.04)
Q3 (10.73–12.18 µg/mL)	382	20	1.53 (0.75–3.15)	1.52 (0.74–3.12)	1.35 (0.66–2.80)	1.43 (0.68–3.00)
Q4 (12.19–24.34 µg/mL)	378	39	3.11 (1.61–6.01)	3.11 (1.61–6.00)	2.70 (1.39–5.24)	2.77 (1.32–5.82)
*P* for trend			0.02	0.02	0.07	0.10
Per 1-SD increment in serum LBP concentration^a^			1.29 (1.08–1.55)	1.29 (1.08–1.55)	1.25 (1.04–1.51)	1.27 (1.00–1.61)

LBP, lipopolysaccharide-binding protein; Q, quartile; SD, standard deviation.

^a^The SD of the serum LBP concentration was 2.2 µg/mL.

^b^Model 1: adjusted for age, sex, current smoking, current drinking, and regular exercise at baseline (2002–2003).

^c^Model 2: adjusted for the covariates in model 1 plus homeostasis model assessment of insulin resistance at baseline.

^d^Model 3: adjusted for the covariates in model 1 plus serum high-sensitivity C-reactive protein at baseline.

In the subgroup analysis of traditional cardiovascular risk factors, moreover, there was no evidence of heterogeneity in the association of serum LBP concentration with the risk of MetS between subgroups of each risk factor (all *P* for heterogeneity ≥0.18) (eTable 3). Then, we examined the association of serum LBP levels with the risk of MetS in the subgroup stratified by sex. As a result, the sensitivity analysis did not change the study conclusion substantially (eTable 4). Also, in the sensitivity analyses using various criteria for MetS, the associations between serum LBP levels and the risk of developing MetS defined by various criteria were substantially similar to the results of the analysis for MetS defined using the Japanese criteria (eTable 5). Furthermore, the findings did not change substantially in the sensitivity analysis after excluding individuals with serum hs-CRP of ≥3.0 mg/L (eTable 6) or individuals with diabetes or with a history of cardiovascular disease at baseline (eTable 7).

Next, we investigated the associations of serum LBP levels with the risk of developing each of the MetS components. As a result, significant associations were observed for the incidence of all components (ie, abdominal obesity, dyslipidemia, elevated blood pressure, and elevated fasting plasma glucose) in the age- and sex-adjusted model and in model 1 (Table 2). However, the associations between serum LBP levels and abdominal obesity were attenuated but remained significant after additional adjustment for HOMA-IR (model 2). Meanwhile, the associations between serum LBP levels and the remaining three components were attenuated to a non-significant level. The associations with the risk of developing all MetS components were not significant after the additional adjustment for serum hs-CRP (model 3).

Finally, we estimated the geometric mean values of HOMA-IR at the follow-up survey in 2007–2008 according to the quartiles of serum LBP levels at the baseline survey in 2002–2003 (Table 3). Elevated serum LBP levels at baseline were significantly associated with higher geometric mean values of HOMA-IR at the follow-up after adjusting for age, sex, baseline HOMA-IR, current smoking, current drinking, and regular exercise (model 2, *P* for trend <0.001). However, when additional adjustment was made for baseline serum hs-CRP, the association was attenuated to a nonsignificant level (model 3, *P* for trend = 0.07).

**Table 3.** Geometric average of HOMA-IR at the follow-up survey in 2007–2008 according to serum LBP levels

Serum LBP levels	Number of participants	Model 1^a^		Model 2^b^		Model 3^c^	

		Geometric average (95% CI)	*P* for trend	Geometric average (95% CI)	*P* for trend	Geometric average (95% CI)	*P* for trend
Q1 (2.20–9.56 µg/mL)	462	1.14 (1.09–1.20)		1.14 (1.09–1.20)		1.17 (1.12–1.23)	
Q2 (9.57–10.78 µg/mL)	471	1.25 (1.19–1.31)		1.25 (1.19–1.31)		1.26 (1.21–1.32)	
Q3 (10.79–12.18 µg/mL)	469	1.25 (1.20–1.31)		1.25 (1.20–1.31)		1.25 (1.19–1.31)	
Q4 (12.19–24.34 µg/mL)	467	1.31 (1.25–1.38)	<0.001	1.31 (1.25–1.37)	<0.001	1.27 (1.20–1.33)	0.07

CI, confidence interval; HOMA-IR, homeostasis model assessment of insulin resistance; LBP, lipopolysaccharide-binding protein; Q, quartile.

^a^Model 1: adjusted for age, sex, and HOMA-IR at baseline (2002–2003).

^b^Model 2: adjusted for the covariates in model 1 plus current smoking, current drinking, and regular exercise at baseline.

^c^Model 3: adjusted for the covariates in model 2 plus serum high-sensitivity C-reactive protein at baseline.

## DISCUSSION

In this prospective study of a general Japanese population, higher serum LBP levels were significantly associated with an increased risk of developing MetS and its components after adjusting for age, sex, and lifestyle factors. However, the additional adjustment for HOMA-IR, an indicator of insulin resistance, attenuated these associations. Furthermore, additional adjustment for serum hs-CRP drastically attenuated these associations to non-significant levels. This association was similar and robust in the sensitivity analysis using different criteria of MetS. The significant positive association between serum LBP and future insulin resistance, represented by the geometric averages of HOMA-IR at the follow-up survey, was also observed in the multivariable-adjusted analysis, but disappeared after additional adjustment for serum hs-CRP. These findings suggest that low-grade endotoxemia is an important risk factor for insulin resistance and subsequent development of MetS through chronic systemic inflammation.

Our findings agree with those from previous cross-sectional studies^11^^,^^12^^,^^20^^,^^21^ that have shown a positive association between serum LBP levels and the presence of MetS and its components, including obesity,^11^^,^^12^^,^^20^ dyslipidemia,^11^^,^^12^ elevated blood pressure,^12^ and elevated plasma glucose.^12^^,^^21^ In addition, there has been only one prospective study conducted in China, which reported that elevated plasma LBP levels were significantly associated with an increased risk of developing MetS and its components, especially hypertriglyceridemia and hyperglycemia, during a 6-year follow-up period.^22^ Our findings, together with previous epidemiological evidence, suggest that individuals with higher serum LBP levels have a greater risk of developing MetS and its components.

Several biological mechanisms could potentially explain the positive association of serum LBP levels with the risk of MetS. Serum LBP forms a complex with lipopolysaccharide absorbed into the bloodstream and binds to TLR4 on the surface of immune cells.^15^ TLR4 activates its own downstream immune response and causes a chronic inflammatory response.^15^^,^^16^ Low-dose intravenous lipopolysaccharide administration to healthy humans was found to induce a rapid and transient induction of plasma TNF-α and IL-6, and mRNA expression of inflammatory cytokines and chemokines in adipose tissue.^30^ These processes are known to modulate adipose and systemic insulin resistance.^9^ Increased insulin resistance due to systemic chronic inflammation has been well recognized to cause various metabolic disorders, including glucose intolerance, dyslipidemia by hyperinsulinemia, and elevated blood pressure through accelerated sodium reabsorption in the kidney.^31^ Intriguingly, the present study showed that the associations of serum LBP levels with the risk of developing MetS and its components—especially dyslipidemia, elevated blood pressure, and elevated fasting plasma glucose—were weakened after adjustment for HOMA-IR and largely attenuated after adjustment for serum hs-CRP. Moreover, the significant positive association of serum LBP levels and future insulin resistance was attenuated after adjusting for serum hs-CRP. These findings may suggest that the associations of serum LBP levels with the risk of developing MetS and its components could be partly explained by the presence of subclinical low-grade inflammation and insulin resistance induced by the lipopolysaccharide–LBP complexes.

The mechanism linking LBP to obesity appears to involve LBP production and activity in adipose tissue and to be bi-directional. Unlike the other three MetS components, central obesity could increase serum LBP, which might exacerbate obesity. LBP is mainly produced by hepatocytes,^10^ but it is also expressed and released by intestinal epithelial cells^32^ and visceral adipocytes.^33^ The amount of intra-abdominal fat has been shown to correlate strongly with the serum concentration of lipopolysaccharide^34^ or LBP,^35^ and the mRNA expression of LBP in adipose tissue and circulating LBP concentration were found to be increased with weight gain and decreased with weight loss.^33^ Although there is a possibility of reverse causality, the present longitudinal study revealed that higher serum LBP levels were associated with greater risk of developing abdominal obesity. Whether low-grade endotoxemia is causally related to the development of abdominal obesity should be validated in further longitudinal studies.

Several limitations in this study should be noted. First, serum LBP levels were measured only at baseline, which did not capture the variability during the follow-up period. Since serum LBP concentrations and other risk factors may have changed, the misclassification of serum LBP levels was possible, which could have led to an underestimation of the association between the serum LBP level and MetS. Second, although we tried to control for a wide range of confounding factors, we could not exclude residual confounding by unmeasured confounders, such as health consciousness^36^ and gut flora.^37^ We cannot rule out the possibility that these factors may be confounding factors that may influence the increase in serum LBP concentration and the risk of developing MetS. Indeed, since information of the stress status and dietary content of residents was not available in the present study, we assumed that there was residual confounding. Third, we could not identify the major organ responsible for the source of increased serum lipopolysaccharide. Since we did not have any information on gut permeability and inflammatory cytokines other than hs-CRP, we were unable to perform a more detailed analysis to clarify the biological mechanism. Fourth, information on prior infections or other condition having presumed acute inflammation was not available in the present study. Prior infections could have caused an acute increase of serum LBP, leading to misclassification of the chronic levels of serum LBP. However, a sensitivity analysis in which individuals with serum hs-CRP of ≥3.0 mg/L were excluded did not make any material difference in the findings. Fifth, there is the possibility of selection bias due to the exclusion of participants from this study due to lack of information. Of 1,459 individuals excluded from the analysis, 815 individuals were excluded from the analysis due to the lack of information at baseline and follow-up surveys, except for 30 individuals without the study consent and the 614 individuals with MetS at baseline who had to be excluded in the longitudinal study design examining the risk of developing outcomes. Therefore, we compared the baseline characteristics between 1,869 individuals included in the analysis and 815 excluded individuals (eTable 8). The excluded individuals were younger, more likely to be men, had lower mean values of systolic and diastolic blood pressure, fasting plasma glucose, and abdominal circumference, higher mean values of serum HDL cholesterol levels, and lower frequencies of use of anti-hypertensive agents than the included individuals. Thus, the excluded population were likely to be a healthier population. Finally, as the participants of the present study were recruited from one town in Japan, we urge caution in generalizing these findings to populations with different genetic backgrounds and lifestyles. Therefore, these results need to be validated by other large-scale, population-based prospective cohort studies in different populations.

### Conclusion

The study demonstrated that elevated serum LBP levels were an important risk factor for the development of MetS in the general Japanese population. These findings emphasize that low-grade endotoxemia may contribute to the pathogenesis of insulin resistance and MetS through chronic systemic inflammation. However, it remains unclear whether serum LBP levels would be an appropriate therapeutic target. Our findings should encourage future studies to clarify the mechanism underlying the association between serum LBP levels and the risk of developing MetS.

## SUPPLEMENTARY MATERIAL

eMaterial 1. Methods

eTable 1. Metabolic syndrome criteria used in the present study

eTable 2. Adjusted direct and indirect associations per 1-SD^a^ increment in serum LBP concentration and metabolic syndrome development mediated via systemic chronic inflammation and insulin resistance

eTable 3. Multivariable-adjusted odds ratios per 1-SD increment in serum LBP concentration for metabolic syndrome by the presence or absence of traditional cardiovascular risk factors

eTable 4. Odds ratios for the development of metabolic syndrome according to serum LBP quartiles by sex

eTable 5. Odds ratios of metabolic syndrome per 1-SD increment in serum LBP concentration

eTable 6. Odds ratios for the development of metabolic syndrome according to serum LBP quartiles, after the exclusion of individuals with serum high-sensitive C-reactive protein of ≥3.0 mg/L (n = 1,474)

eTable 7. Odds ratios for the development of metabolic syndrome according to quartiles of serum LBP levels among individuals without a history of diabetes and/or cardiovascular disease at baseline (n = 1,619)

eTable 8. Baseline characteristics of individuals included and excluded from the present study, the Hisayama Study, 2002

eFigure 1. Selection process of the examined population for analyses of each component of metabolic syndrome. HDLC, high-density lipoprotein cholesterol; LBP, lipopolysaccharide-binding protein; TG, triglycerides.
