# Supplementary material for: Serum Lipopolysaccharide-binding Protein Levels and the Incidence of Metabolic Syndrome in a General Japanese Population: the Hisayama Study
Source: J Epidemiol. 2024 Jan 5;34(1):1–7. doi: 10.2188/jea.JE20220232 (PMC10701254; doi:10.2188/jea.JE20220232)
Supplement: Supplementary file 1 [file je-34-001-s001.pdf]

eMaterial 1. Methods

The detailed criteria of metabolic syndrome proposed by the Japanese Society of Internal Medicine (Japanese criteria)[1]

In the criteria of metabolic syndrome proposed by the Japanese Society of Internal Medicine, abdominal obesity (waist circumference ≥85 cm for men and ≥90 cm for women) is an essential component for diagnosis of MetS. MetS was defined as having abdominal obesity and the presence of at least two of the following three components: (1) dyslipidemia (serum triglycerides ≥1.69 mmol/L and/or serum HDL cholesterol <1.03 mmol/L and /or current use of lipid-modifying agents), (2) elevated blood pressure (blood pressure ≥130/85 mm Hg and/or current use of anti-hypertensive agents), and (3) elevated fasting plasma glucose (fasting plasma glucose ≥6.1 mmol/L and/or current use of glucose-lowering agents).

Clinical evaluation and laboratory measurements

At the baseline and follow-up examinations, each participant completed a self-administered questionnaire covering medical history, treatment for hypertension, diabetes mellitus, and dyslipidemia, current smoking, current drinking, and regular exercise. The questionnaire was checked by trained interviewers. Current smoking and current drinking were classified into currently habitual or not. The individuals engaging in sports or other forms of exertion ≥3 times a week during their leisure time made up a regular exercise group. The waist circumference was measured at the umbilical level in a standing position by a trained staff member. Body height and weight were measured in light clothing without shoes, and body mass index was calculated (kg/m^2). Blood pressure was measured 3 times using an automated sphygmomanometer in the sitting position after a rest of at least 5 minutes. The average of three measurements was used for analysis. Hypertension was defined as

a blood pressure ≥140/90 mm Hg and/or current use of anti-hypertensive agents. Blood samples were collected from an antecubital vein after an overnight fast for the determination of serum lipids and plasma glucose levels. Serum total cholesterol, high-density lipoprotein (HDL) cholesterol, and triglyceride levels were measured enzymatically. Plasma glucose levels were measured by the hexokinase method, and serum insulin levels were determined by a commercial double antibody solid-phase radioimmunoassay (Phadeseph Insulin; Pharmacia Diagnostics AB). Diabetes mellitus was determined by a fasting glucose level ≥7.0 mmol/L, a 2-hour post-load glucose level after 75-g oral glucose tolerance test ≥11.1 mmol/L (94.7% of the examined population [n=1,770] received a 75-g oral glucose tolerance test) and/or current use of glucose-lowering agents (ie, oral hypoglycemic agents or insulin) according to the WHO 1998 guideline.[2] Insulin resistance was evaluated by homeostasis model assessment of insulin resistance (HOMA-IR) values,[3] which were calculated as follows: HOMA-IR = fasting plasma glucose (mmol/L) × fasting serum insulin (IU/mL) / 22.5. Serum hs-CRP concentrations were measured using a modification of the Behring Latex-Enhanced CRP assay on a BN-100 Nephelometer (Behring Diagnostics, Westwood, MA) from frozen samples.

REFERENCES

1. Committee to Evaluate Diagnostic Standards for Metabolic Syndrome. Definition and the diagnostic standard for metabolic syndrome [in Japanese]. *Nihon Naika Gakkai Zasshi*. 2005;94:794-809.

2. Alberti KGMM, Zimmet PZ. Definition, diagnosis and classification of diabetes mellitus and its complications. Part 1 Diagnosis and classification of diabetes mellitus

Provisional report of a WHO consultation. *Diabet Med.* 1998;15:539-553. Doi: 10.1002/(SICI)1096-9136(199807)15:7<539: :AID-DIA668>3.0.CO;2-S

3. Matthews DR, Hosker JP, Rudenski AS, Naylor BA, Treacher DF, Turner RC. Homeostasis model assessment: insulin resistance and beta-cell function from fasting plasma glucose and insulin concentrations in man. *Diabetologia*. 1985;28:412-419. Doi: 10.1007/BF00280883

eTable 1. Metabolic syndrome criteria used in the present study

	A. Japanese	B. Modified Japanese	C. IDF	D. EGIR	E. Joint statement from IDF/NHLBI/AHA/WHF/IAS/IASO
Definition of metabolic syndrome	Component (1) below, plus any two or more of components (2)–(4)	Component (1) below, plus any two or more of components (2)–(4)	Component (1) below, plus any two or more of components (2)–(4)	Insulin resistance (defined as hyperinsulinaemia, in turn defined as the top 25% of fasting insulin values among those with non-diabetes mellitus) plus two of the following	Three or more of the following
Components					
Abdominal obesity (waist circumference)	(1) ≥85 cm (men), ≥90 cm (women)	(1) ≥90 cm (men), ≥80 cm (women)	(1) ≥90 cm (men), ≥80 cm (women)	(1) ≥94 cm (men), ≥80 cm (women)	(1) ≥90 cm (men), ≥80 cm (women)
Dyslipidemia	(2) serum triglycerides ≥1.69 mmol/L and/or serum HDLC <1.03 mmol/L and/or lipid-modifying agents	(2) Same as A	(2) serum triglycerides ≥1.69 mmol/L and/or lipid-modifying agents<br>(3) serum HDLC <1.03 mmol/L (men), <1.29 mmol/L (women) and/or lipid-modifying agents	(2) serum triglycerides >2.0 mmol/L and/or serum HDLC <1.0 mmol/L and/or lipid-modifying agents	(2) Same as C<br>(3) Same as C
Elevated blood pressure	(3) ≥130/85 mm Hg and/or antihypertensive agents	(3) Same as A	(4) Same as A	(3) ≥140/80 mm Hg and/or antihypertensive agents	(4) Same as A
Elevated fasting plasma glucose	(4) ≥6.1 mmol/L and/or glucose-lowering agents	(4) Same as A	(5) ≥5.6 mmol/L and/or glucose-lowering agents	(4) ≥6.1 mmol/L but non-diabetes mellitus	(5) ≥5.6 mmol/L and/or glucose-lowering agents

AHA, American Heart Association; EGIR, European Group for the Study of Insulin Resistance; HDLC, high-density lipoprotein cholesterol; IAS, International Atherosclerosis Society; IASO, International Association for the Study of Obesity; IDF, International Diabetes Federation; NHLBI, National Heart, Lung, and Blood Institute; WHF, World Heart Federation.

eTable 2. Adjusted direct and indirect associations per 1-SD[a] increment in serum LBP concentration and metabolic syndrome development mediated via systemic chronic inflammation and insulin resistance

Mediator	Odds ratios [b]	(95% confidence intervals)	*P* value
HOMA-IR			
Total association	1.39	(1.15–1.63)	0.001
Direct association	1.25	(1.04–1.46)	0.02
Indirect association via HOMA-IR	1.11	(1.06–1.16)	<0.001
Proportion mediated, %	35.0		
Serum hs-CRP			
Total association	1.35	(1.14–1.55)	0.001
Direct association	1.06	(0.85–1.27)	0.55
Indirect association via serum hs-CRP	1.27	(1.12–1.42)	<0.001
Proportion mediated, %	81.7		

LBP, lipopolysaccharide-binding protein; HOMA-IR, homeostasis model assessment of insulin resistance; CRP, C-reactive protein; SD, standard deviation.
[a]The SD of the serum LBP concentration was 2.2 μg/mL.
[b] Adjusted for age, sex, current smoking, current drinking, and regular exercise.

eTable 3. Multivariable-adjusted odds ratios per 1-SD increment in serum LBP concentration for metabolic syndrome by the presence or absence of traditional cardiovascular risk factors

Variables [c]		Persons at risk	Number of events	Odds ratio (95% confidence interval) per 1-SD increment in serum LBP concentration [a b]	*P* for heterogeneity
Overall		1,869	159	1.37 (1.17–1.60)	
Age	<65 years	1,183	94	1.36 (1.12–1.66)	0.63
	≥65 years	686	65	1.37 (1.05–1.79)	
Sex	Men	686	84	1.25 (0.99–1.58)	0.18
	Women	1,183	75	1.46 (1.19–1.79)	
Hypertension	No	1,195	77	1.32 (1.05–1.65)	0.92
	Yes	674	82	1.34 (1.07–1.68)	
Diabetes mellitus	No	1,669	112	1.32 (1.10–1.58)	0.75
	Yes	200	47	1.47 (1.03–2.11)	
Body mass index	<25 kg/m^2	1,502	76	1.33 (1.07–1.65)	0.31
	≥25 kg/m^2	367	83	1.13 (0.87–1.47)	
Current smoking	No	1,484	122	1.37 (1.14–1.63)	0.97
	Yes	385	37	1.39 (1.01–1.92)	
Current drinking	No	1,062	82	1.46 (1.19–1.78)	0.37
	Yes	807	77	1.25 (0.99–1.59)	
Regular exercise	No	1,670	140	1.34 (1.14–1.58)	0.68
	Yes	199	19	1.76 (1.07–2.89)	

LBP, lipopolysaccharide-binding protein; SD, standard deviation.

[a] The SD of the serum LBP concentration was 2.2 μg/mL.

[b] The model was adjusted for age, sex, current smoking, current drinking, and regular exercise at baseline (2002–2003).

[c] The variables relevant to the subgroup were excluded from the corresponding model.

eTable 4. Odds ratios for the development of metabolic syndrome according to serum LBP quartiles by sex

Serum LBP levels	Persons at risk	Number of events	Odds ratio (95% confidence interval)			
			Age- and sex-adjusted	Model 1 [b]	Model 2 [c]	Model 3 [d]
**Metabolic syndrome (Men)**						
Q1 (5.17–9.68 μg/mL)	171	14	1.00 (reference)	1.00 (reference)	1.00 (reference)	1.00 (reference)
Q2 (9.70–11.00 μg/mL)	171	20	1.56 (0.76–3.22)	1.51 (0.73–3.12)	1.28 (0.61–2.67)	1.40 (0.67–2.90)
Q3 (11.03–12.36 μg/mL)	173	25	2.03 (1.01–4.09)	1.94 (0.96–3.91)	1.56 (0.76–3.19)	1.56 (0.75–3.25)
Q4 (12.37–22.75 μg/mL)	171	25	2.14 (1.05–4.34)	2.20 (1.08–4.49)	1.72 (0.83–3.57)	1.50 (0.67–3.37)
P for trend			0.03	0.02	0.12	0.32
Per 1-SD increment in serum LBP concentration [a]			1.24 (0.99–1.55)	1.25 (0.99–1.57)	1.17 (0.93–1.49)	1.04 (0.79–1.37)
**Metabolic syndrome (Women)**						
Q1 (2.20–9.49 μg/mL)	295	5	1.00 (reference)	1.00 (reference)	1.00 (reference)	1.00 (reference)
Q2 (9.51–10.66 μg/mL)	290	18	3.80 (1.39–10.38)	3.87 (1.41–10.59)	3.06 (1.10–8.52)	3.11 (1.13–8.57)
Q3 (10.67–12.02 μg/mL)	302	27	5.55 (2.09–14.69)	5.57 (2.10–14.78)	3.66 (1.36–9.86)	3.81 (1.41–10.29)
Q4 (12.03–24.34 μg/mL)	296	25	5.17 (1.93–13.84)	5.16 (1.93–13.80)	2.95 (1.07–8.10)	2.23 (0.76–6.60)
P for trend			< 0.001	< 0.001	0.08	0.30
Per 1-SD increment in serum LBP concentration [a]			1.47 (1.20–1.81)	1.46 (1.19–1.80)	1.30 (1.04–1.63)	1.07 (0.81–1.40)

LBP, lipopolysaccharide-binding protein; Q, quartile; SD, standard deviation.
[a] The SD of the serum LBP concentration was 2.1 μg/mL for men and 2.2 μg/mL for women.
[b] Model 1: adjusted for age, sex, current smoking, current drinking, and regular exercise at baseline (2002–2003).
[c] Model 2: adjusted for the covariates in model 1 plus homeostasis model assessment of insulin resistance at baseline.
[d] Model 3: adjusted for the covariates in model 1 plus serum high-sensitivity C-reactive protein at baseline.

eTable 5. Odds ratios of metabolic syndrome per 1-SD increment in serum LBP concentration

Criteria	Persons at risk	Number of events	Age- and sex-adjusted		Model 1 [b]		Model 2 [c]		Model 3 [d]	
			Odds ratio [a] (95% CI)	*P* value	Odds ratio [a] (95% CI)	*P* value	Odds ratio [a] (95% CI)	*P* value	Odds ratio [a] (95% CI)	*P* value
Japanese	1,869	159	1.37 (1.17–1.59)	< 0.001	1.37 (1.17–1.60)	< 0.001	1.25 (1.06–1.48)	0.008	1.06 (0.87–1.30)	0.54
Modified Japanese	1,872	190	1.28 (1.11–1.48)	0.001	1.29 (1.12–1.49)	0.001	1.19 (1.02–1.39)	0.03	1.05 (0.88–1.26)	0.60
IDF	1,686	230	1.25 (1.09–1.43)	0.001	1.26 (1.10–1.44)	0.001	1.18 (1.02–1.36)	0.03	0.99 (0.83–1.17)	0.87
EGIR	2,037	90	1.28 (1.05–1.56)	0.01	1.28 (1.05–1.56)	0.01	1.17 (0.95–1.45)	0.14	1.13 (0.87–1.45)	0.37
Joint statement from IDF/NHLBI/AHA/WHF /IAS/ IASO	1,529	246	1.15 (1.00–1.31)	0.05	1.15 (1.01–1.32)	0.01	1.07 (0.92–1.23)	0.40	0.97 (0.82–1.15)	0.70

AHA, American Heart Association; CI, confidence interval; EGIR, European Group for the Study of Insulin Resistance; IAS, International Atherosclerosis Society; IASO, International Association for the Study of Obesity; IDF, International Diabetes Federation; LBP, lipopolysaccharide-binding protein; NHLBI, National Heart, Lung, and Blood Institute; SD, standard deviation; WHF, World Heart Federation.

[a] The SD of the serum LBP concentration was 2.2 μg/mL.

[b] Model 1: adjusted for age, sex, current smoking, current drinking, and regular exercise at baseline (2002–2003).

[c] Model 2: adjusted for the covariates in model 1 plus homeostasis model assessment of insulin resistance at baseline.

[d] Model 3: adjusted for the covariates in model 1 plus serum high-sensitivity C-reactive protein at baseline.

eTable 6. Odds ratios for the development of metabolic syndrome according to serum LBP quartiles, after the exclusion of individuals with serum high-sensitive C-reactive protein of ≥3.0 mg/L (n=1,474)

Serum LBP levels	Persons at risk	Number of events	Odds ratio (95% confidence interval)			
			Age- and sex-adjusted	Model 1 [b]	Model 2 [c]	Model 3 [d]
**Metabolic syndrome**						
Q1 (2.20–9.46 μg/mL)	434	13	1.00 (reference)	1.00 (reference)	1.00 (reference)	1.00 (reference)
Q2 (9.47–10.64 μg/mL)	437	37	3.02 (1.58–5.79)	3.03 (1.58–5.80)	2.58 (1.34–4.98)	2.42 (1.25–4.68)
Q3 (10.65–11.90 μg/mL)	437	48	4.04 (2.14–7.62)	4.06 (2.15–7.66)	3.07 (1.61–5.84)	2.69 (1.39–5.18)
Q4 (11.92–17.77 μg/mL)	436	47	3.79 (2.00–7.19)	3.80 (2.00–7.21)	2.57 (1.33–4.95)	2.00 (1.00–3.97)
P for trend			< 0.001	< 0.001	0.01	0.17
Per 1-SD increment in serum LBP concentration [a]			1.45 (1.22–1.72)	1.45 (1.22–1.73)	1.31 (1.09–1.57)	1.16 (0.95–1.41)

LBP, lipopolysaccharide-binding protein; Q, quartile; SD, standard deviation.

[a] The SD of the serum LBP concentration was 1.9 μg/mL.

[b] Model 1: adjusted for age, sex, current smoking, current drinking, and regular exercise at baseline (2002–2003).

[c] Model 2: adjusted for the covariates in model 1 plus homeostasis model assessment of insulin resistance at baseline.

[d] Model 3: adjusted for the covariates in model 1 plus serum high-sensitivity C-reactive protein at baseline.

eTable 7. Odds ratios for the development of metabolic syndrome according to quartiles of serum LBP levels among individuals without a history of diabetes and/or cardiovascular disease at baseline (n=1,619)

Serum LBP levels	Persons at risk	No. of events	Odds ratio (95% confidence interval)			
			Age- and sex-adjusted	Model 1 [b]	Model 2 [c]	Model 3 [d]
**Metabolic syndrome**						
Q1 (2.20–9.46 μg/mL)	402	11	1.00 (reference)	1.00 (reference)	1.00 (reference)	1.00 (reference)
Q2 (9.47–10.67 μg/mL)	406	27	2.66 (1.30–5.47)	2.67 (1.30–5.50)	2.33 (1.12–4.4)	2.55 (1.38–4.71)
Q3 (10.68–12.09 μg/mL)	400	32	3.24 (1.60–6.68)	3.27 (1.61–6.63)	2.52 (1.23–4.83)	2.61 (1.41–4.84)
Q4 (12.10–24.34 μg/mL)	411	38	3.68 (1.84–7.41)	3.66 (1.82–7.36)	2.73 (1.34–4.82)	2.18 (1.12–4.24)
P for trend			< 0.001	< 0.001	0.01	0.07
Per 1-SD increment in serum LBP concentration [a]			1.32 (1.16–1.58)	1.32 (1.09–1.58)	1.23 (1.01–1.48)	1.06 (0.87–1.30)

LBP, lipopolysaccharide-binding protein; Q, quartile; SD, standard deviation.

[a] The SD of the serum LBP concentration was 2.2 μg/mL.

[b] Model 1: adjusted for age, sex, current smoking, current drinking, and regular exercise at baseline (2002–2003).

[c] Model 2: adjusted for the covariates in model 1 plus homeostasis model assessment of insulin resistance at baseline.

[d] Model 3: adjusted for the covariates in model 1 plus serum high-sensitivity C-reactive protein at baseline.

eTable 8. Baseline characteristics of individuals included and excluded from the present study, the Hisayama Study, 2002

	Included in the study (n=1,869)	Excluded from the study [a] (n=815)	*P* value
Age, years, mean (SD)	64.8 (12.1)	53.4 (9.5)	<0.001
Men, %	36.6	41.5	0.02
Hypertension, %	43.2	24.4	<0.001
Use of anti-hypertensive agents, %	26.0	10.4	<0.001
Systolic blood pressure, mean (SD)	131 (20)	125 (19)	<0.001
Diastolic blood pressure, mean (SD)	77 (11)	76 (11)	0.01
Diabetes mellitus, %	14.1	9.9	0.003
Fasting plasma glucose, mmol/L, mean (SD)	2.8 (0.6)	2.7 (0.4)	0.04
Serum total cholesterol, mmol/L, mean (SD)	5.2 (0.9)	5.3 (0.9)	0.22
Serum HDL cholesterol, mmol/L, mean (SD)	1.6 (0.4)	1.7 (0.5)	0.002
Serum triglyceride, mmol/L, median (interquartile range)	2.4 (1.8–3.3)	2.3 (1.7–3.4)	0.77
Body mass index, kg/m^2, mean (SD)	22.4 (3.0)	22.5 (3.0)	0.72
Waist circumference, cm, mean (SD)	80.5 (8.5)	79.2 (8.4)	<0.001
Current smoking, %	17.0	29.7	<0.001
Current drinking, %	37.8	49.9	<0.001
Regular exercise, %	11.7	9.3	0.07
HOMA-IR, median (interquartile range)	1.5 (1.1–2.3)	1.6 (1.1–2.3)	0.89
Serum hs-CRP, mg/L, median (interquartile range)	0.4 (0.2–1.0)	0.4 (0.2–0.8)	0.17

HDL, high-density lipoprotein; HOMA-IR, homeostasis model assessment of insulin resistance; hs-CRP, high-sensitivity C-reactive protein; IQR, interquartile range; LBP, lipopolysaccharide-binding protein; SD, standard deviation.

[a] The number of excluded participants is shown as the maximum value. Each value was calculated from the available data, excluding missing values.

FIGURE LEGENDS

eFigure 1. Selection process of the examined population for analyses of each component of metabolic syndrome. HDLC, high-density lipoprotein cholesterol; LBP, lipopolysaccharide-binding protein; TG, triglycerides.

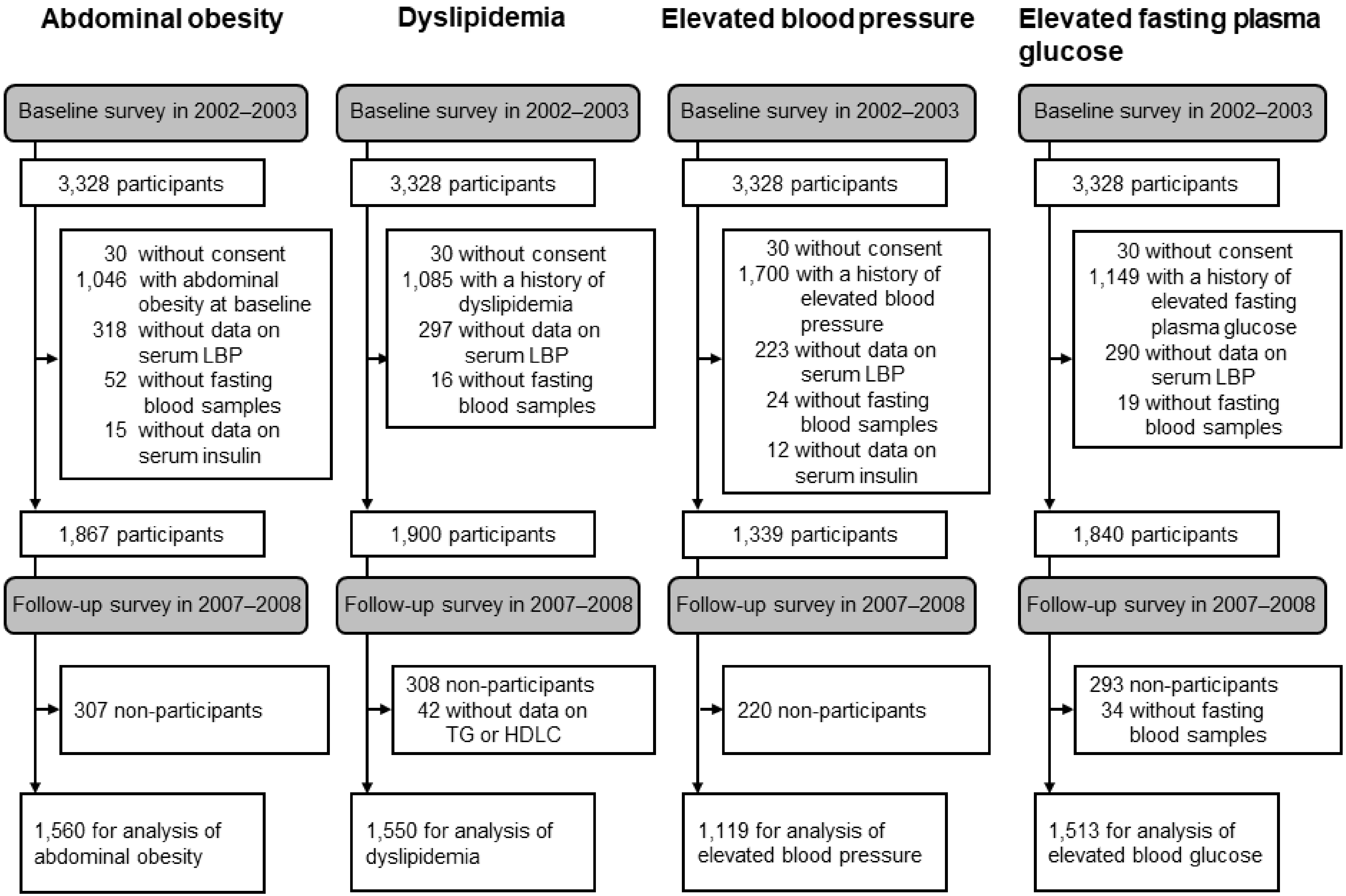
Abdominal obesity
Dyslipidemia
Elevated blood pressure
Elevated fasting plasma glucose
Baseline survey in 2002–2003
3,328 participants
30 without consent
1,046 with abdominal obesity at baseline
318 without data on serum LBP
52 without fasting blood samples
15 without data on serum insulin
1,867 participants
Follow-up survey in 2007–2008
307 non-participants
1,560 for analysis of abdominal obesity
30 without consent
1,085 with a history of dyslipidemia
297 without data on serum LBP
16 without fasting blood samples
1,900 participants
308 non-participants
42 without data on TG or HDLC
1,550 for analysis of dyslipidemia
30 without consent
1,700 with a history of elevated blood pressure
223 without data on serum LBP
24 without fasting blood samples
12 without data on serum insulin
1,339 participants
220 non-participants
1,119 for analysis of elevated blood pressure
30 without consent
1,149 with a history of elevated fasting plasma glucose
290 without data on serum LBP
19 without fasting blood samples
1,840 participants
293 non-participants
34 without fasting blood samples
1,513 for analysis of elevated blood glucose
